# A positive psychology perspective on self-efficacy and work engagement among psychiatric hospital staff in Japan: A cross-sectional study

**DOI:** 10.1038/s41598-026-52413-x

**Published:** 2026-05-20

**Authors:** Kazumi Kubota, Ayumi Sugimura, Satoko Fujihara, Saeko Ishibashi, Satoko Tsuda, Makoto Fujiki, Shiho Hayashi, Tokuma Kino

**Affiliations:** 1 Shimonoseki City University, 2-1-1 Daigaku-cho, Shimonoseki, 751-8510 Yamaguchi Japan; 2 National Center for Child Health and Development, Setagaya-ku, Tokyo, Japan; 3 Department of Healthcare Information Management, The University of Tokyo Hospital, Bunkyo-ku, Tokyo, Japan; 4 Faculty of Nursing, Gifu University, Gifu, Gifu Japan; 5 Institute for Health Economics and Policy, Minato-ku, Tokyo, Japan; 6 School of Nursing, Sapporo City University, Sapporo, Hokkaido Japan; 7 College of Life and Health Sciences, Chubu University, Kasugai, Aichi Japan; 8 Holy Cross Hospital (Seijuji Hospital), Toki, Gifu Japan

**Keywords:** Positive psychology, Work engagement, Self-efficacy, Psychological capital, Job Demands–Resources model, Healthcare workers, Psychiatric hospital, Japan, Health care, Health occupations, Psychology, Psychology, Risk factors

## Abstract

Sustaining work engagement among psychiatric hospital staff is important for staff well-being and the quality and continuity of mental health care, yet modifiable personal resources in this setting remain understudied. Guided by the Job Demands–Resources model and positive psychology, we examined whether general self-efficacy was associated with work engagement in psychiatric hospital staff in Japan and whether this association differed across occupation, managerial position, and age. In a cross-sectional survey of 199 employees, work engagement was assessed using the Japanese Utrecht Work Engagement Scale (UWES-J-17) and self-efficacy using the Japanese General Self-Efficacy Scale (J-GSES). Linear regression models estimated the association between self-efficacy and engagement, adjusting for gender, age group, managerial position, employment status, and occupation; subgroup consistency was evaluated using stratified analyses and interaction terms. Higher self-efficacy was associated with higher work engagement (B = 0.93, 95% CI 0.42–1.44, *p* < 0.001). The association appeared similar across occupational categories, managerial levels, and age groups (all interaction *p* ≥ 0.43). In this single-site sample, general self-efficacy was positively associated with work engagement. Causal inference is not possible, and longitudinal and intervention studies are needed.

**Supplementary Information:**

The online version contains supplementary material available at 10.1038/s41598-026-52413-x.

## Introduction

Work engagement is a positive, fulfilling work-related state characterized by vigor, dedication, and absorption, and is associated with employee well-being as well as important care-related outcomes in healthcare^1, 2, 3, 4^. In psychiatric services, maintaining engagement may be particularly important because staff often work under sustained emotional demands, complex therapeutic relationships, safety concerns, and coordination across inpatient, outpatient, and community-based care^5, 6^. Identifying modifiable factors associated with engagement in this setting is therefore relevant not only to worker well-being but also to the quality and continuity of mental health care.

Self-efficacy, defined by Bandura as beliefs in one’s capabilities to organize and execute actions required to manage prospective situations, is associated with persistence, goal setting, coping, and resilience^7, 8, 9^. The Job Demands–Resources (JD–R) model provides a useful framework for understanding why self-efficacy may be relevant to work engagement. In this model, jobs involve demands that require sustained effort and resources that can support motivation and well-being. In addition to job-based resources, the model also recognizes personal resources—positive self-evaluations linked to a sense of control and effectiveness^10, 11^. Self-efficacy is one such personal resource and may help employees cope with demands, make use of available resources, and remain energetically invested in their work. Prior cross-sectional and longitudinal studies in various occupational settings have reported positive associations between self-efficacy and engagement and have suggested possible gain cycles in which efficacy beliefs, positive affect, and engagement reinforce one another over time^12, 13, 14, 15, 16^.

Important gaps remain. Much of the existing literature has focused on single professions, non-psychiatric settings, or Western contexts, leaving it unclear whether the association between self-efficacy and work engagement is evident across the diverse occupational groups that comprise psychiatric hospitals, across hierarchical levels, and across career stages. This question may be especially relevant in Japan, where psychiatric care has gradually shifted toward community-oriented models and where organizational culture and collectivist work norms may shape how personal resources relate to engagement^17, 18^. Examining these issues in a Japanese psychiatric hospital may therefore offer both contextual and practical value.

In the present study, we focused on general self-efficacy rather than task- or profession-specific efficacy. This choice was intentional because psychiatric hospitals employ a heterogeneous workforce spanning clinical, welfare/support, administrative, and other roles, and our aim was to examine whether a broadly shared personal resource was associated with engagement across this workforce using a common metric. At the same time, domain-specific efficacy beliefs—such as confidence in managing psychiatric symptoms, de-escalating aggression, or coordinating mental health care—may be more proximal to some aspects of work engagement in psychiatric practice. Against this background, this study examined the association between general self-efficacy and work engagement among employees of a Japanese psychiatric hospital and tested whether this association was consistent across occupational categories, managerial position, and age groups. We hypothesized that higher self-efficacy would be associated with higher engagement independent of demographics and occupation, and that this association would be broadly similar across subgroups.

We examined the personal-resources aspect of the JD–R model by testing the association between general self-efficacy and work engagement. Because we did not directly measure job demands or job resources, the study should be interpreted as examining one theoretically relevant component of the JD–R framework rather than testing the full model.

## Subjects and methods

### Study design and participants

We conducted a cross-sectional, anonymous questionnaire survey at a mid-sized psychiatric hospital in Gifu Prefecture, Japan, in September 2020. All hospital employees on payroll at the time of the survey (*N* = 350) were eligible, regardless of occupation, managerial status, or employment status. Paper questionnaires and an explanatory letter were distributed via internal mail. The letter described the study purpose, voluntary participation, confidentiality, and anonymity. Completed questionnaires were returned in sealed envelopes to a secure collection point. Returning a complete questionnaire indicated consent; non-return indicated decline without consequences. No incentives were provided.

Of 350 questionnaires, 217 were returned (62.0%). We excluded 18 with missing data on key variables (work engagement, self-efficacy, or covariates), yielding 199 complete responses for analysis (effective response rate 56.9%). A priori power calculation was not performed due to the census approach; post hoc, the sample provided > 90% power to detect small-to-moderate associations (standardized beta ≥ 0.20) at alpha 0.05.

### Measures

#### Work engagement

Work engagement was assessed using the Japanese Utrecht Work Engagement Scale (UWES-J-17; 17 items, 0–6 Likert, total 0–102; higher scores indicate greater engagement)^19, 20^. The UWES captures vigor, dedication, and absorption and has demonstrated acceptable validity in Japanese working populations.^19, 20^ Internal consistency in the present sample was excellent (Cronbach’s alpha 0.94), and score distributions showed minimal floor or ceiling effects.

#### Self-efficacy

General self-efficacy was assessed using the Japanese General Self-Efficacy Scale (J-GSES; 16 dichotomous yes/no items, total 0–16; higher scores indicate greater self-efficacy)^20, 21, 22^. The J-GSES was selected because the study aimed to assess a common personal resource across a heterogeneous hospital workforce using a single metric applicable to clinical, welfare/support, administrative, and other roles. The scale has been used as a measure of generalized efficacy beliefs and was appropriate for the present hospital-wide, cross-occupational research question. Internal consistency in the present sample was good (Cronbach’s alpha 0.85), and floor or ceiling effects were minimal. We recognize that domain-specific efficacy measures may be more proximal to particular psychiatric-care tasks; accordingly, our inferences are limited to generalized efficacy beliefs rather than task-specific competence.

#### Demographics and work characteristics

Participants reported gender (male, female), age group (20–29, 30–39, 40–49, 50 or older), managerial position (non-management, management defined as supervisor level or above), employment status (full-time, part-time), and occupation. The original 15 occupations were pooled into 10 analytic categories: physician, registered nurse, practical nurse, nursing assistant, mental health social worker, clerical staff, dietitian (registered dietitian or dietitian), cook (licensed cook or kitchen staff), childcare worker, and other (occupational therapist, psychologist, laboratory technologist, or miscellaneous). For stratified analyses, occupations were grouped a priori into three categories: healthcare professionals (physicians, registered/practical nurses, nursing assistants), welfare or support staff (mental health social workers, childcare workers), and administrative or other personnel (clerical, dietitians, cooks, other).

#### Data management and quality control

Data were double-entered independently by two team members and cross-checked. Range and logic checks were performed for all variables. Missingness was low and not systematically associated with observed covariates; listwise deletion was used for analyses requiring complete cases. To further protect confidentiality in this single-site study, very small occupational categories were aggregated for reporting, and the study site is described in general terms in the main manuscript.

#### Statistical analysis

Descriptive statistics summarized sample characteristics. The primary analysis used linear regression with work engagement (UWES total) as the dependent variable and self-efficacy (J-GSES total) as the independent variable.

Model 1 (unadjusted) estimated the bivariate association between self-efficacy and engagement. Model 2 (adjusted) included gender, age group, managerial position, employment status, and occupation as covariates, selected a priori based on the JD-R model and prior literature^10, 11^. Categorical predictors were dummy-coded with references: male (gender), 20–29 years (age), non-management (position), full-time (employment), and physician (occupation).

To examine the consistency of associations across subgroups (i.e., potential effect modification), we assessed interaction terms in the full sample and presented unadjusted stratified simple regressions for interpretability. Specifically, we tested: (1) self-efficacy × occupational category (healthcare, welfare/support, administrative/other); (2) self-efficacy × managerial position (non-managers vs. managers), and additionally compared coefficients between groups with a coefficient-difference z test (Fisher’s Z transformation); and (3) self-efficacy × age group (20–39 vs. ≥ 40 years). Non-significant interactions were interpreted as no statistical evidence of effect modification, acknowledging the limited power of interaction tests.

Where institutional denominator data were available, we compared the distribution of respondents with that of the source workforce descriptively using available category distributions for broad observable characteristics. Because denominator data were limited and were not available in sufficient detail for all subgroupings, this comparison was intended as a pragmatic descriptive check rather than a formal statistical test of non-response bias. We assessed whether there were any large discrepancies in the broad available distributions; no marked differences were apparent, although non-response bias cannot be ruled out.

We report unstandardized coefficients (B), standardized coefficients (beta), 95% confidence intervals, p-values, and model fit (R², adjusted R²). Two-tailed alpha was 0.05. Analyses used SPSS Version 28.0 (IBM Corp., Armonk, NY, USA).

## Results

### Participant characteristics

Of 199 participants, 63 were male (31.7%) and 136 female (68.3%). Age groups were 20–29 years 36 (18.1%), 30–39 years 60 (30.2%), 40–49 years 41 (20.6%), and 50 or older 62 (31.2%). Most held non-management positions (170, 85.4%) and were full-time (169, 84.9%). The sample included a range of clinical, welfare/support, administrative, and other support occupations. To protect confidentiality, occupational categories were aggregated for reporting. The largest group was registered nurses (88, 44.2%), followed by welfare/support staff (25, 12.6%) and practical nurses/nursing assistants (22, 11.1%) (Table 1).

**Table 1:** Participant characteristics (*n* = 199).

Characteristics	*n* (%)	Mean ± SD
Gender		
Male	63 (31.7)	
Female	136 (68.3)	
**Age**		
20 to 29	36 (18.1)	
30 to 39	60 (30.2)	
40 to 49	41 (20.6)	
≥ 50	62 (31.2)	
**Position**		
Non-management	170 (85.4)	
Management	29 (14.6)	
**Employment Status**		
Full-time	169 (84.9)	
Part-time	30 (15.1)	
**Occupation**		
Physician	17 (8.5)	
Registered Nurse	88 (44.2)	
Practical Nurse/Nursing Assistant	22 (11.1)	
Welfare/Support Staff	25 (12.6)	
Clerical Staff	16 (8.0)	
Nutrition/Food Service Staff	12 (6.0)	
Allied Health/Other Support Staff	19 (9.5)	
Work Engagement*		44.82 ± 16.18
Self-Efficacy**		5.91 ± 4.17

Notes: Occupational categories were aggregated for reporting to protect confidentiality in this single-site study. For regression analyses, occupations were coded as described in the Methods. Higher scores indicate greater work engagement and self-efficacy.

### Association between self-efficacy and work engagement

In unadjusted analysis, self-efficacy was positively associated with engagement (B = 1.602, 95% CI 1.105–2.098, β = 0.413, *p* < 0.001; R² = 0.171; F(1,197) = 40.49, *p* < 0.001). In adjusted analysis, self-efficacy remained significant (B = 0.928, 95% CI 0.419–1.437, β = 0.239, *p* < 0.001). The full adjusted model explained 36.1% of variance (adjusted R² = 0.305; F(16,182) = 6.43, *p* < 0.001). An increase of one SD in self-efficacy (4.17 points) corresponded to approximately 3.9 points higher engagement (0.24 SD).

Managerial position was associated with substantially higher engagement (B = 10.593, 95% CI 4.763–16.422, *p* < 0.001). Compared with physicians, registered nurses (B = − 9.517, *p* = 0.021), practical nurses (B = − 11.464, *p* = 0.026), and nursing assistants (B = − 12.492, *p* = 0.047) reported lower engagement; other occupations did not differ significantly. Gender, age group, and employment status were not significant in adjusted models. Multicollinearity was acceptable (all variance inflation factors < 5; range 1.31–4.49)^23^. See Table 2.

**Table 2:** Linear regression analysis: unadjusted and adjusted models (*N* = 199).

Variable	Model 1				Model 2			
	B	β	95% CI	*p*	B	β	95% CI	*p*
Self-Efficacy	1.602	0.413	1.105 to 2.098	< 0.001	0.928	0.239	0.419 to 1.437	< 0.001
Gender (Ref: Male)		-						
Female		-			2.458	0.071	−2.166 to 7.081	0.296
Age group (Ref: 20–29 years)		-						
30–39 years		-			1.669	0.047	−4.190 to 7.528	0.575
40–49 years		-			5.446	0.136	−1.042 to 11.933	0.099
≥ 50 years		-			5.161	0.148	−1.442 to 11.763	0.125
Managerial position (Ref: Non-management)								
Management		-			10.593	0.232	4.763 to 16.422	< 0.001
Employment status (Ref: Full-time)								
Part-time		-			2.567	0.057	−3.747 to 8.881	0.424
Occupation (Ref: Physician)		-						
Registered Nurse		-			−9.517		−17.569 to −1.465	0.021
Practical Nurse		-			−11.464		−21.549 to −1.379	0.026
Nursing Assistant		-			−12.492		−24.789 to −0.194	0.047
Mental Health Social Worker		-			−0.464		−10.705 to 9.776	0.929
Clerical Staff		-			−1.263		−11.197 to 8.671	0.802
Dietitian		-			−3.776		−15.823 to 8.271	0.537
Cook		-			−12.865		−30.571 to 4.842	0.153
Childcare Worker		-			3.911		−7.727 to 15.549	0.508
Other		-			6.078		−3.061 to 15.217	0.191

Notes. B = unstandardized regression coefficient; β = standardized partial regression coefficient; CI = confidence interval; Ref = reference. Model 1 presents unadjusted bivariate associations. Model 2 adjusted for gender, age group, managerial position, employment status, and occupation. Reference categories: male, age 20–29 years, non-management, full-time, physician (occupation). All tests two-tailed with α = 0.05. Model fit statistics: Model 1: R² = 0.17, adjusted R² = 0.17, F(1, 197) = 40.49, *p* < 0.001; Model 2: R² = 0.36, adjusted R² = 0.31, F(16, 182) = 6.43, *p* < 0.001.

### Consistency of associations across subgroups

Interaction terms were non-significant unless otherwise noted. Self-efficacy predicted engagement consistently across occupational categories: healthcare professionals (B = 1.58, 95% CI 0.94–2.22, *p* < 0.001, R² 0.167), welfare or support staff (B = 1.54, 95% CI 0.31–2.77, *p* = 0.016, R² 0.154), and administrative or other personnel (B = 1.72, 95% CI 0.76–2.68, *p* = 0.001, R² 0.180). The interaction test was non-significant (*p* = 0.917) (Table 3).

**Table 3:** Stratified analysis: occupational categories.

Category	*n*	B	95% CI	*p*	*R*²
Healthcare	127	1.58	0.94 to 2.22	< 0.001	0.167
Welfare/support	25	1.54	0.31 to 2.77	0.016	0.154
Administrative/other	47	1.72	0.76 to 2.68	0.001	0.18
Interaction test				0.917	

Notes: B = self-efficacy coefficient from simple linear regression predicting work engagement; CI = confidence interval; R² = proportion of variance explained. Interaction test: self-efficacy × category interaction p-value.

By managerial level, self-efficacy predicted engagement among non-managers (B = 1.48, 95% CI 0.89–2.07, *p* < 0.001, R² 0.148) and managers (B = 2.14, 95% CI 0.56–3.72, *p* = 0.010, R² 0.324), with no significant coefficient difference (Fisher’s Z *p* = 0.435) (Table 4).

**Table 4:** Stratified analysis: hierarchical levels.

Level	*n*	B	95% CI	*p*	*R*²
Non-managers	170	1.48	0.89 to 2.07	< 0.001	0.148
Managers	29	2.14	0.56 to 3.72	0.010	0.324
Coefficient difference†		-	-	0.435	-

Notes: B = unstandardized self-efficacy coefficient from simple linear regression predicting work engagement within each managerial level; CI = confidence interval; R² = proportion of variance explained. † Coefficient difference test: p-value from Fisher’s Z transformation comparing regression coefficients between groups. All stratified analyses are unadjusted (simple linear regression).

By age, self-efficacy predicted engagement among younger staff (20–39 years; B = 1.78, 95% CI 0.98–2.58, *p* < 0.001, R² 0.205) and older staff (40 or older; B = 1.44, 95% CI 0.68–2.20, *p* < 0.001, R² 0.142), with a non-significant interaction (*p* = 0.437) (Table 5).

**Table 5:** Stratified analysis: age groups.

Age group	*n*	B	95% CI	*p*	*R*²
Younger (20–39 years)	96	1.78	0.98 to 2.58	< 0.001	0.205
Older (≥ 40 years)	103	1.44	0.68 to 2.20	< 0.001	0.142
Interaction test				0.437	

Notes: B = unstandardized self-efficacy coefficient from simple linear regression predicting work engagement within each age group. CI = confidence interval; R² = proportion of variance explained. Interaction test p-value from regression model including self-efficacy × age group interaction term. All stratified analyses are unadjusted (simple linear regression).

### Sensitivity analyses

Excluding registered nurses (*n* = 111), the adjusted association remained significant (B = 0.91, 95% CI 0.24–1.58, *p* = 0.008; adjusted R² 0.287). Restricting analyses to full-time employees (*n* = 169) yielded similar results (B = 0.98, 95% CI 0.42–1.54, *p* = 0.001; adjusted R² 0.318). No extreme outliers (± 3 SD) or influential observations (Cook’s distance > 4/n) were detected (Table 6).

**Table 6:** Sensitivity analyses.

	*n*	Unadjusted			Adjusted		
		B	95% CI	*p*	B	95% CI	*p*
Excluding RNs	111	1.52	0.82 to 2.22	< 0.001	0.91	0.24 to 1.58	0.008
Full-time only	169	1.62	1.04 to 2.20	< 0.001	0.98	0.42 to 1.54	0.001

Notes: RN = registered nurse. B = unstandardized regression coefficient; CI = confidence interval. Adjusted models control for gender, age, position, employment, occupation. No outliers (± 3 SD) or influential cases (Cook’s D > 4/n) detected.

## Discussion

### Principal findings

In this diverse psychiatric hospital workforce, higher self-efficacy was associated with higher work engagement independent of measured demographics and occupation. The association appeared broadly similar across occupational categories, hierarchical levels, and age groups within this sample, although subgroup analyses should be interpreted cautiously given limited power for interaction testing.

### Theoretical implications

Results support the JD-R model’s proposition that personal resources like self-efficacy bolster motivational processes leading to engagement^10, 11^. The observed effect size aligns with prior evidence linking efficacy beliefs to engagement and suggests the potential for gain cycles and resource caravans^12, 13, 14, 15, 16^. The cross-occupational, cross-hierarchy, and cross-age consistency indicates that self-efficacy functions as a broadly applicable personal resource.

### Managerial role and structural resources

Managers’ substantially higher engagement is consistent with literature on enriched work design, autonomy, influence, and recognition^24, 25^. Similar self-efficacy–engagement slopes across hierarchical levels imply that building efficacy may benefit all staff, while structural factors typical of managerial roles likely explain the larger mean-level advantage^26, 27^. Extending autonomy, voice, and development opportunities beyond managerial roles may boost engagement at scale^28, 29^.

### Occupational disparities and nursing

Lower engagement among nursing staff aligns with evidence of high demands, exposure to violence, emotional labor, staffing constraints, and recognition challenges in psychiatric nursing^5, 6, 30, 31, 32, 33, 34^. Addressing these disparities will require both individual-level strategies (e.g., efficacy-building) and system-level reforms (e.g., staffing, safety, resources, career development)^35, 36^.

### Implications for positive psychology interventions

Building on positive psychology and the JD–R model, organization-wide initiatives to strengthen self-efficacy may be promising.^7 ^Scalable components can target Bandura’s four sources of efficacy: mastery experiences, vicarious learning, social persuasion, and regulation of affective states^7^. Practical examples include brief mastery-based skills labs tailored to role demands, structured peer mentoring/shadowing, strengths-based supervisory feedback routines, and stress-management or mindfulness micro-sessions.^37, 38, 39, 40^ A pragmatic evaluation (e.g., stepped-wedge or cluster-randomized rollout across units) could assess feasibility, fidelity, and changes in self-efficacy and engagement over time.

### Practical implications

Managers’ higher mean engagement, independent of self-efficacy, underscores the importance of structural job resources. Extending autonomy, participation in decision-making, recognition, and development pathways to frontline staff could amplify engagement gains^28, 29, 41, 42^. Given the cross-occupational consistency of associations, such organizational changes can be deployed at scale with role-specific tailoring. For nursing, targeted actions around staffing adequacy, safety, resources, recognition, and career progression remain priorities alongside efficacy-building^35, 36, 43^.

### Limitations

This study has several limitations. First, the cross-sectional design precludes causal inference. Although the JD–R framework and prior longitudinal research support the plausibility of self-efficacy as a personal resource related to engagement, reverse causation and reciprocal associations remain possible. In addition, both self-efficacy and work engagement were assessed by self-report, which raises the possibility of common method bias.

Second, we did not measure job demands or job resources directly. Accordingly, the study should be interpreted as a partial test of one JD–R proposition rather than a test of the full model. Residual confounding by unmeasured factors such as workload, supervisor support, organizational climate, and personality traits is also possible.

Third, statistical power for subgroup interaction tests was limited, and some occupational groups were small. To protect confidentiality and maintain interpretability, very small occupational categories were aggregated in reporting. The observed non-significant interactions therefore suggest, but do not prove, similarity of associations across subgroups. In addition, although descriptive comparison with available workforce denominator data did not suggest substantial differences in the broad observable characteristics that could be compared, this was not a formal assessment of non-response bias across all subgroupings, and such bias cannot be ruled out.

Finally, this was a single-site study conducted during the COVID-19 period, which limits generalizability. The organizational context of one psychiatric hospital in Japan may not represent other hospitals or non-pandemic conditions. In addition, because we assessed general rather than domain-specific self-efficacy, our findings pertain to generalized efficacy beliefs across the workforce and do not address psychiatric-care-specific competencies. Multi-site longitudinal studies using both general and task-specific efficacy measures, alongside direct measures of job demands and resources, would strengthen the evidence base.

## Conclusions

In this cross-sectional single-site study, general self-efficacy was positively associated with work engagement among psychiatric hospital staff, and the association appeared similar across occupational, hierarchical, and age subgroups within this sample. These findings are consistent with the JD–R proposition that personal resources may relate to engagement, but they should be interpreted cautiously given the study design. If supported by longitudinal and intervention research, efforts to strengthen self-efficacy alongside structural workplace improvements—particularly for nursing staff—may help support engagement in psychiatric care settings.

### Reporting summary

Further information on research design is available in the Reporting Summary file.

## Supplementary Information

Below is the link to the electronic supplementary material.

Supplementary Material 1

## Data Availability

De-identified aggregate data and analysis code are available from the corresponding author upon reasonable request and subject to institutional ethics approval and a data use agreement. Requests should include a brief proposal and institutional ethics approval (or a waiver). Following approval by the Seijuji Hospital Clinical Research Ethics Committee, we will provide (i) de-identified aggregate tables sufficient to reproduce the main analyses and (ii) SPSS syntax used to generate results, under a Data Use Agreement. Individual-level data cannot be publicly shared due to privacy considerations in a single-institution sample.
