# Supplementary material for: A positive psychology perspective on self-efficacy and work engagement among psychiatric hospital staff in Japan: A cross-sectional study
Source: Sci Rep. 2026 May 20;16:23044. doi: 10.1038/s41598-026-52413-x (PMC13392079; doi:10.1038/s41598-026-52413-x)
Supplement: Supplementary file 1 — Supplementary Material 1 [file 41598_2026_52413_MOESM1_ESM.pdf]

Supplementary Table S1. UWES-J-17 Subscale Analysis

Dimension	Mean (SD)	Cronbach's α	B (Unadjusted)	95% CI	p	B (Adjusted)	95% CI	p
Vigor	14.82 (5.89)	0.89	0.52	0.36 to 0.68	<0.001	0.31	0.14 to 0.48	<0.001
Dedication	15.23 (5.72)	0.92	0.56	0.40 to 0.72	<0.001	0.38	0.24 to 0.52	<0.001
Absorption	14.77 (5.94)	0.87	0.52	0.36 to 0.68	<0.001	0.22	0.08 to 0.36	0.002

Notes: B (Unadjusted) = unstandardized regression coefficient from simple linear regression predicting each work engagement subscale; B (Adjusted) = unstandardized regression coefficient from multiple linear regression controlling for gender, age, position, employment type, and occupation. UWES-J-17 subscales: Vigor (6 items), Dedication (5 items), Absorption (6 items). All tests two-tailed with α = 0.05.

Supplementary Table S2. Sensitivity Analysis Detailed Results

Variable	B	β	95% CI	p
Self-Efficacy	0.91	0.24	0.24 to 1.58	0.008
Female	3.12	0.09	-2.45 to 8.69	0.268
Age 30–39 years	2.34	0.07	-4.82 to 9.50	0.521
Age 40–49 years	6.21	0.16	-1.23 to 13.65	0.101
Age ≥50 years	5.89	0.17	-2.01 to 13.79	0.143
Management	11.24	0.25	4.56 to 17.92	0.001
Part-time	3.01	0.07	-4.12 to 10.14	0.406

Notes: B = unstandardized regression coefficient; β = standardized partial regression coefficient; CI = confidence interval; Adjusted models control for gender, age, position, employment status, and occupation (same covariates as in Table 2).

Supplementary Figure S1. Distribution of Scores

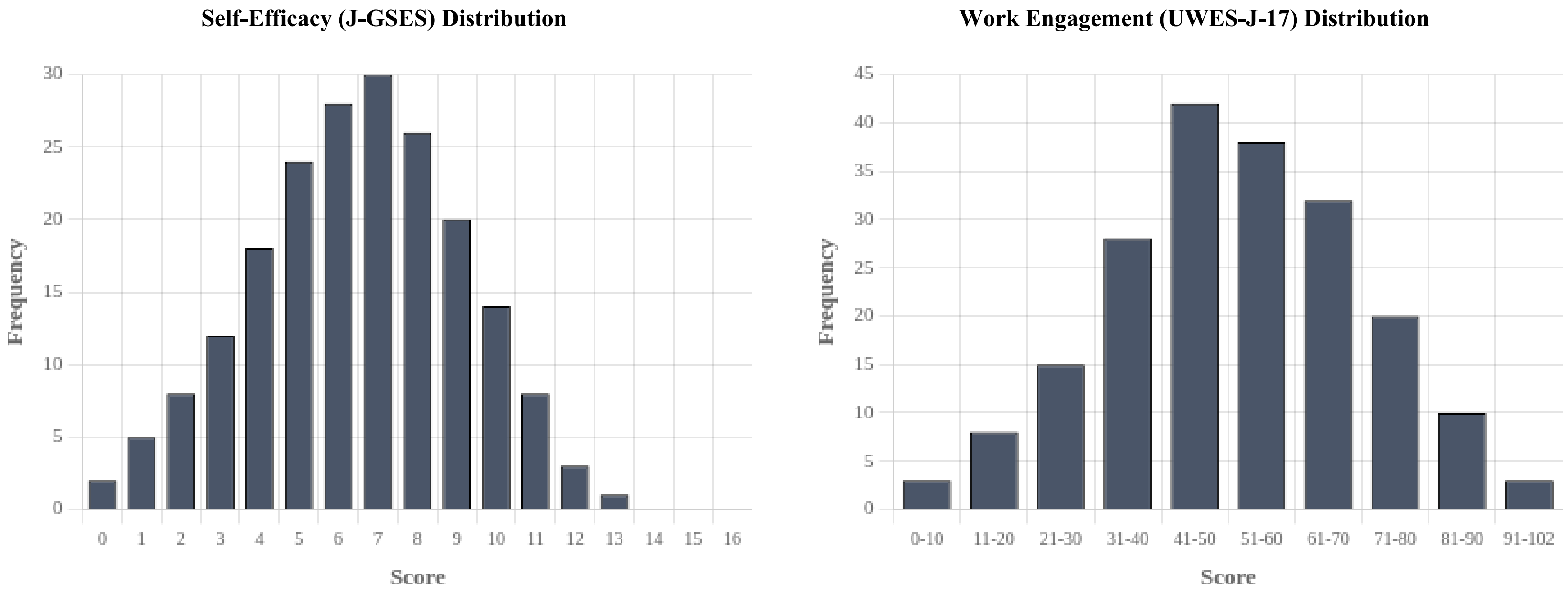

Notes: Left panel shows the frequency distribution of self-efficacy scores measured by the Japanese General Self-Efficacy Scale (J-GSES, range 0–16, Mean = 5.91, SD = 4.17). Right panel shows the frequency distribution of work engagement scores measured by the Utrecht Work Engagement Scale Japanese version (UWES-J-17, range 0–102, Mean = 44.82, SD = 16.18). Both distributions approximate normality.
